# A Simple Method to Assess *In Vivo* Proliferation in Lung Vasculature with EdU: The Case of MMC-Induced PVOD in Rat

**DOI:** 10.1155/2015/326385

**Published:** 2015-08-09

**Authors:** Antigny Fabrice, Ranchoux Benoît, Nadeau Valérie, Edmund Lau, Bonnet Sébastien, Perros Frédéric

**Affiliations:** ^1^Faculté de Médecine, Université Paris-Sud, Le Kremlin-Bicêtre, France; ^2^AP-HP, Centre de Référence de l'Hypertension Pulmonaire Sévère, Département Hospitalo-Universitaire (DHU) Thorax Innovation, Service de Pneumologie et Réanimation Respiratoire, Hôpital de Bicêtre, Le Kremlin-Bicêtre, France; ^3^UMRS 999, INSERM and Université Paris-Sud, Laboratoire d'Excellence (LabEx) en Recherche sur le Médicament et l'Innovation Thérapeutique (LERMIT), Centre Chirurgical Marie Lannelongue, Le Plessis-Robinson, France; ^4^Groupe de Recherche en Hypertension Pulmonaire, Centre de Recherche de l'Institut Universitaire de Cardiologie et de Pneumologie de Québec, QC, Canada

## Abstract

5-Ethynyl-2′-deoxyuridine (EdU) incorporation is becoming the gold standard method for *in vitro* and *in vivo* visualization of proliferating cells. The small size of the fluorescent azides used for detection results in a high degree of specimen penetration. It can be used to easily detect DNA replication in large tissue samples or organ explants with low proliferation and turnover of cells formerly believed to be in a “terminal” state of differentiation. Here we describe a protocol for the localization and identification of proliferating cells in quiescent or injured pulmonary vasculature, in a model of pulmonary veno-occlusive disease (PVOD). PVOD is an uncommon form of pulmonary hypertension characterized by progressive obstruction of small pulmonary veins. We previously reported that mitomycin-C (MMC) therapy is associated with PVOD in human. We demonstrated that MMC can induce PVOD in rats, which currently represents the sole animal model that recapitulates human PVOD lesions. Using the EdU assay, we demonstrated that MMC-exposed lungs displayed areas of exuberant microvascular endothelial cell proliferation which mimics pulmonary capillary hemangiomatosis, one of the pathologic hallmarks of human PVOD. *In vivo* pulmonary cell proliferation measurement represents an interesting methodology to investigate the potential efficacy of therapies aimed at normalizing pathologic angioproliferation.

## 1. Introduction

In 2008, Salic and Mitchison [1] developed a method to detect DNA synthesis in proliferating cells based on the incorporation of 5-ethynyl-2′-deoxyuridine (EdU) and its subsequent detection by a fluorescent azide through a Cu(I)-catalyzed [3 + 2] cycloaddition reaction (‘‘click” chemistry). This method was revolutionary because detection of the EdU label is highly sensitive and can be accomplished in minutes. EdU is a nucleoside analog of thymidine that is incorporated into DNA during active DNA synthesis, similar to 5-bromodeoxyuridine (BrdU). In contrast to the BrdU assay, the EdU method does not require harsh chemical or enzymatic disruption of helical DNA structure to enable direct measurement of cells in the S-phase. Elimination of this requirement results in the preservation of helical DNA structure and other cell surface epitopes, preservation of morphological structure, and increased reproducibility [2]. The small size of the fluorescent azides used for detection results in a high degree of specimen penetration, allowing the staining of whole-mount preparations of large tissue and organ explants. This method has been tested using several cell lines and is preferred over traditional BrdU detection since it is more sensitive and allows multicolor and multiplex analysis in flow cytometry and imaging [3]. Hence, this method can be used to quantify proliferation in cultured cells as well as organs of whole animals [1]. For these reasons, the use of EdU in animals is growing and has been reported in experimental models of neoplastic diseases [4–11], cardiovascular and metabolic diseases [12–15], and neuroimmunologic development [13, 15, 16].

The normal endothelium is usually considered to be a genetically stable, quiescent cell line [17]. This means that there are very low proliferation and turnover of endothelial cells (EC). In this normal condition, it can be difficult to measure proliferation with methods that utilize the detection of proteins associated with cell cycle activation (such as PCNA, Ki67) at a single time point (i.e., the time of animal sacrifice or tissue sampling). In contrast,* in vivo* labeling of DNA-duplicating proliferating cells with EdU over hours or even days allows the detection of rare events of proliferation. For this reason we used this technique to track EC proliferation in controls and in animals affected by pulmonary veno-occlusive disease (PVOD).

PVOD is a rare and devastating cause of pulmonary hypertension (PH). Idiopathic or familial pulmonary arterial hypertension (PAH) is characterized by major remodeling of precapillary small pulmonary arteries (<500 *μ*m) with typical complex lesions, plexiform lesions, while PVOD preferentially affects the postcapillary venous pulmonary vessels [18–20]. PVOD shares many similarities with PAH, from risk factors to clinical or hemodynamic presentation, which can easily lead to misdiagnosis between these two conditions. However, PVOD is characterized by a poor prognosis and the possibility of developing severe pulmonary edema with specific PAH therapy. Pathologic hallmarks of PVOD include fibrous intimal proliferation of septal veins and preseptal venules in association with capillary proliferation within the alveolar septa (also known as pulmonary capillary hemangiomatosis).

However, the pathophysiologic mechanisms of PVOD remain poorly understood, particularly due to the lack of PVOD animal models and few studies have addressed the pathobiology of this very rare disease in humans [21]. PVOD may be heritable due to biallelic mutations in the* EIF2AK4* gene that codes for general control nonderepressible 2 protein (GCN2) [22]. Drug exposures can also induce PVOD and a recent study from the French PH network suggests that chemotherapeutic alkylating agents can trigger the development of PVOD [23, 24]. Some cases of severe PVOD have been reported after therapy with mitomycin-C (MMC). In line with these observations, we recently confirmed a causal link between MMC and human PVOD and developed a model of PVOD in rats induced by MMC administration [25].

In rats, MMC exposure induced preferential remodeling of pulmonary venous and capillary compartments, representing currently the sole animal model of PVOD. MMC-induced PVOD in rats was also characterized by pronounced capillary proliferation within the alveolar septa mimicking human condition.

## 2. Material and Methods

### 2.1. Ethics

Rats were housed at the Faculty of Pharmacy of Châtenay-Malabry (ANIMEX platform, Châtenay-Malabry, France). Experiments were conducted according to the European Union regulations (Directive 86/609 EEC) for animal experiments and complied with our institution's guidelines for animal care and handling. The animal facility is licensed by the French Ministry of Agriculture (agreement number B92-019-01). This study was approved by the Committee on the Ethics of Animal Experiments CEEA26 CAP Sud. Animal experiments were supervised by Dr. Frederic Perros (agreement delivered by the French Ministry of Agriculture for animal experiment number A92–392). All efforts were made to minimize animal suffering.

### 2.2. MMC-Induced PVOD

PVOD was induced in female Wistar rat (100 g) by MMC, as previously described [25].

### 2.3. *In Vivo* Cell Proliferation Assay

To evaluate cell proliferation in rats, we visualized the incorporation of exogenously supplied 5-ethynyl-2′-deoxyuridine (EdU), to identify cells undergoing DNA replication.

(3.1) Inject EdU intraperitoneally into the rats at 50 mg/kg, 24 h before sacrifice.
(3.2) After hemodynamic measurements, exsanguinate the rat by aortic puncture. Then, inflate the lungs* via* the trachea with a cryoprotector medium (10.24% alcohol polyvinyl, 4.26% polyethylene glycol) diluted in PBS (1 : 1). Ligate the trachea and flash-freeze the lungs in 2-methylbutane precooled over dry ice.
(3.3) Cut 6 *μ*m lung section using a cryotome and air dry.
(3.4) Fix dried lung sections with paraformaldehyde (PFA) 4% for 10 minutes at room temperature and quench free aldehyde groups from PFA fixation with 50 mM NH_4_Cl solution.
(3.5) Permeabilize with Triton 3% and saturate the tissue with human (10%) and rat (10%) serum in PBS for 1 hour at room temperature.
(3.6) Detect EdU using the click-iT EdU imaging kit (C10337, Life Technologies, Saint-Aubin, France) according to the manufacturer's instructions.
(3.7) Perform double immunostaining against endothelial cell markers, fibroblast, and smooth muscle cell markers. Pulmonary fibroblast cells can be identified using antibody against Vimentin-TRITC (Santa Cruz Biotechnology clone V9), pulmonary microvascular endothelial cells can be labelled using antibody against CD34 (Clone QBEnd 10, Dako), and smooth muscle cells can be labelled using antibody against *α*-SMA (clone 1A4) as previously described by Ranchoux and collaborators [26].
(3.8) Analyse the lung sections under a fluorescence microscope by quantifying the number of EdU-positive cells.

### 2.4. Histology

Five *μ*m sections of paraffin-embedded rat lung tissue were stained with Masson's trichrome as previously described [27].

## 3. Representative Results

### 3.1. MMC Induces Pulmonary Vascular Remodeling in the Rat, with Pulmonary Venous and Capillary Lesions Mimicking Capillary Hemangiomatosis Found in PVOD Patients

**Figure 1:**
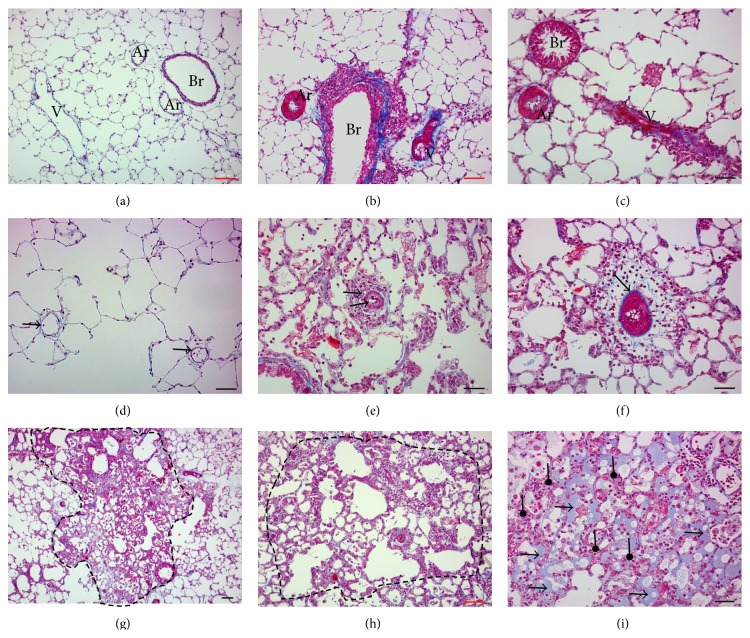
MMC induces pulmonary vascular remodeling in the rat, with pulmonary venous and capillary lesions mimicking capillary hemangiomatosis found in PVOD patients. ((a) and (d)) Control lung. ((b)-(c), (e)–(i)) MMC-exposed lung. (a) Control pulmonary artery (Ar) and vein (V) close to the bronchus (Br). ((b)-(c)) Remodeling of pulmonary arteries and veins close to the bronchi at two levels of distality. (d) Control distal microvessels (arterioles and venules are indistinguishable) (arrows). (e) Remodeled distal pulmonary arteriole delimited by apparent internal and external elastica (blue stained) (arrow). (f) Remodeled distal pulmonary venule delimited by single elastica (blue stained) (arrow). ((g)-(h)) Foci of alveolar-wall thickening suggestive of pulmonary capillary hemangiomatosis (dotted line area). (i) Foci of pulmonary edema (arrow) and capillaritis (arrowhead). ((a)–(i)) Masson's trichrome stained paraffin-embedded tissue sections: muscle fibers (red), collagen (blue), light red/pink (cytoplasm), and cell nuclei (dark brown/black). Red scale bar = 100 *μ*m. Black scale bar = 50 *μ*m.

Recently, we described the first relevant animal model of PVOD induced by MMC exposure [25]. We depict here a fine analysis of the MMC-induced PVOD lesions stained with Masson's trichrome. Compared to the control arteries and veins (Figure 1(a)), we observed severe pulmonary vascular remodeling in rats with MMC-induced PVOD. This remodeling was characterized by smooth muscle cell hypertrophy/hyperplasia in pulmonary arteries and intimal thickening of pulmonary veins (Figures 1(b) and 1(c)). We also observed neomuscularization and adventitial inflammation of the small distal arterioles and venules (Figures 1(e) and 1(f)) which are normally not muscularized in control rats (Figure 1(d)). There were foci of alveolar-wall thickening suggestive of pulmonary capillary hemangiomatosis (Figures 1(g) and 1(h)). Finally we observed foci of pulmonary oedema and capillaritis (Figure 1(i)). The venular remodeling with foci of alveolar-wall thickening, pulmonary oedema, and capillaritis observed in MMC rats is very similar to the PVOD lesions observed in human patient lungs.

### 3.2. MMC-Exposed Rat Lungs Display Areas of Intense Microvascular Endothelial Cell Proliferation

**Figure 2:**
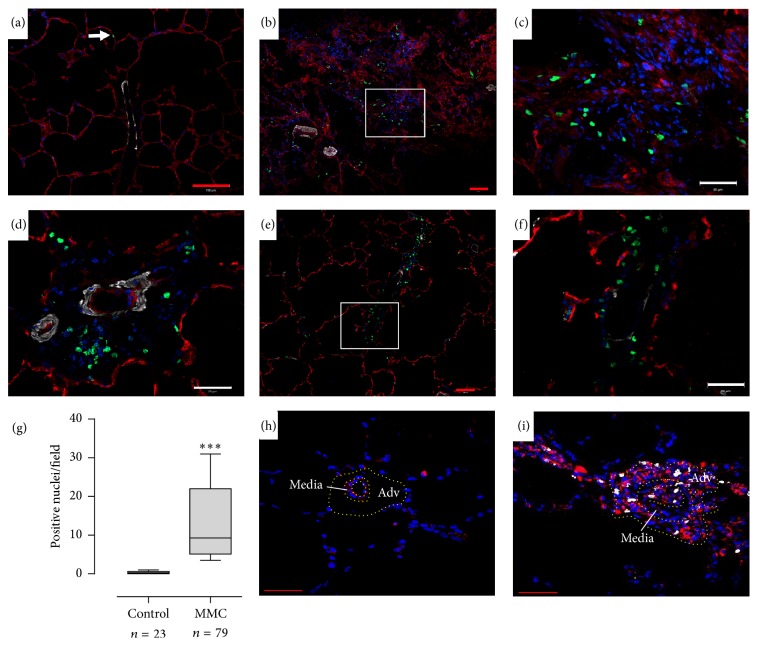
MMC-exposed rat lungs display areas of intense microvascular endothelial cell proliferation. Immunofluorescent staining of frozen rat lung sections and confocal imaging. Red: CD34 (endothelial cells). White: *α*-smooth muscle actin (smooth muscle cells). Green: click-iT EdU stain (nuclei of proliferating cells). Counterstain = 4′,6-diamidino-2-phenylindole (DAPI). (a) Control rat lung. ((b)–(f)) MMC-exposed rat lung. (a) Control rat lung parenchyma displays very little microvascular endothelial cell proliferation. Note a single endothelial cell in proliferation (arrow). ((b)-(c)) Pulmonary capillary hemangiomatosis like foci from MMC-exposed rats display intense microvascular endothelial cell proliferation. (d) Adventitial fibroblast cells proliferation in small remodeled pulmonary artery. ((e)-(f)) Adventitial fibroblast cells proliferation in small remodeled pulmonary vein. (g) Quantification of the percentage of lung proliferating cells. ((h)-(i)) Exuberant adventitial fibroblast cell proliferation in MMC-exposed rats (i) compared to control rats (h) (Vimentin+EdU+ cells). Adventitial fibroblast cells were stained with antibody against Vimentin (fibroblast marker in red) and proliferative cells with click-it EdU (white nuclei = EdU positive nuclei = proliferating cells). Vessel media and adventitia (Adv) were delimited by the yellow dotted line area. ^*∗∗∗*^ *P* < 0.001. Red scale bar = 100 *μ*m. White scale bar = 50 *μ*m.

In order to examine cell proliferation in rats, we visualized the incorporation of exogenous EdU to identify cells undergoing DNA replication. As shown in Figure 2, we observed very few EdU-positive cells in the control lung parenchyma (Figure 2(a)). In MMC-exposed lungs, we observed numerous foci of intense microvascular endothelial cell proliferation (Figures 2(b) and 2(c)) (parenchymal CD34+EdU+ cells). We also observed adventitial cell proliferation in the remodeled pulmonary microvessels (Figures 2(d)–2(f)). Using co-immunostaining against fibroblast marker (Vimentin, in red, (Figures 2(h) and 2(i))) and EdU (white nuclei = proliferative cells), we confirmed the abnormal proliferation of adventitial fibroblast cells (Vimentin positive cells) in MMC-exposed rats (Figure 2(h)) compared to control rat (Figure 2(i)). The overall cellular proliferation in the lung was significantly increased (Figure 2(g)) (*P* < 0.001). These observations confirm an active proliferation of microvascular endothelial cells and fibroblast in the observed lesions. These lesions are similar to PVOD lesion observed in patient lungs and provide confirmation of MMC-exposed rats as a promising animal model of human PVOD.

## 4. Discussion

Cell proliferation analyses are crucial for the assessment of altered cell growth and cell differentiation, which is reminiscent of many diseases including PAH, PVOD, cancers, systemic hypertension, and arteriosclerosis. Cell proliferation analyses are also useful to evaluate the toxicity or/and the potential efficacy of therapeutic molecules. We recently demonstrated that two chemotherapeutic drugs (MMC and cyclophosphamide) could induce PVOD in rats (Figure 1). PVOD is primarily due to progressive occlusion of the pulmonary postcapillary bed due to the aberrant proliferation of vascular cells. Using EdU in combination with classical immunostaining, we were able to easily identify the proliferating cell populations [25]. As illustrated in Figure 2, MMC-exposed lungs displayed areas of intense microvascular endothelial cell proliferation, mimicking capillary hemangiomatosis found in human PVOD. We also observed adventitial fibroblast cell proliferation in the remodeled pulmonary microvessels of these animals. These observations highlight the crucial role of microvascular endothelial cells and adventitial fibroblasts in the pathogenesis of PVOD.* In vivo* pulmonary cell proliferation measurement with EdU staining represents an important technique to investigate the potential efficacy of therapeutic interventions aimed at reversing pathologic angioproliferation.

Compared with EdU incorporation, a major drawback of the BrdU staining method is the requirement for a DNA denaturation step, typically by hydrochloric acid treatment or by heating, in order to expose the incorporated BrdU to the anti-BrdU antibody. These harsh staining conditions can damage tissue structure and potentially destroy cellular epitopes [1, 2]. With our EdU incorporation protocol, we were easily able to perform triple staining, adding immunofluorescent staining for *α*-SMA, CD34, and Vimentin, in addition to EdU staining.

In our study, EdU was injected intraperitoneally into the rats at 50 mg/kg, 24 h before sacrifice. However, depending on the cell type, organ of interest, or animal species of the disease model and other parameters, the protocol will need to be refined for optimal assessment of cell proliferation. Some authors have found that the number of EdU (+) cells was associated with EdU concentration, incubation time, and the volume of click reaction solution [28]. For instance, Guo et al. [12] used EdU in a model of neointimal formation after catheter balloon injury in GK and Wistar rats. EdU stainings for GK rats were performed at an increasing dose of 5 mg/kg, 25 mg/kg, 50 mg/kg, 100 mg/kg, and 200 mg/kg (i.p.). Better staining results were obtained with multiple EdU injections at 100 mg/kg and no better results were obtained with 200 mg/kg. In the adult nervous system, Zeng et al. [29] found that the EdU dose-response data showed that the EdU-labeled cell numbers slightly increased as the EdU dose increased from 10 to 200 mg/kg (i.p.). A 50 mg/kg dose of EdU resulted in near saturation labeling of proliferating cells in the dentate gyrus of the hippocampus. Amano et al. also gave EdU to mice in drinking water containing 1 mg/mL EdU [13]. Refinement of the time necessary to label proliferating cells* in vivo* is also required. For instance, in organs with high cell turnover such as the intestine, a short duration of labeling (2 hours) was sufficient to designate a baseline position of proliferating crypt cells [30]. In some cases, users have to pay attention to the potential toxicity of this method. Indeed, Andersen et al. [31] demonstrated that stem cell survival was severely compromised after EdU incorporation even at low EdU concentrations.

EdU can also be used to track proliferating cell. Salic and Mitchison [1] used EdU to image the cellular turnover in small intestine villi. EdU staining could be detected strongly in cells present at the base of the intestinal villi, which is the location of the most actively dividing cells. Ninety-six hours after the EdU pulse, the labeled cells have multiplied and migrated distally, away from the base of the villi toward their tips [1]. Similarly, Asano et al. [32] used dual labeling with BrdU and EdU for the estimation of cell migration rate in the small intestinal epithelium. Cell migration rate was calculated by dividing the distance between the median cell positions of the distribution of BrdU- and EdU-positive cells by the time between the injection of BrdU and EdU. Using this dual labeling technique, they estimated approximately 9 and 5 *μ*m/h for the cell migration rates on the villi in the jejunum and ileum, respectively [32]. Hence, the combination of BrdU and EdU, meanwhile, offers a simple and robust way to perform double-DNA labeling. Zeng et al. [29] used this double staining for tracking two populations of neurons generated at different time points. Finally, Salic and Mitchison [1] stated that EdU should facilitate high-resolution electron microscopy studies of cellular chromatin. The EdU-labeling method would indeed be well suited for optical imaging of cellular DNA at nanometer resolution.

## References

[1] Salic A., Mitchison T. J. (2008). A chemical method for fast and sensitive detection of DNA synthesis *in vivo*. *Proceedings of the National Academy of Sciences of the United States of America*.

[2] Buck S., Bradford J., Gee K., Agnew B., Clarke S., Salic A. (2008). Detection of S-phase cell cycle progression using 5-ethynyl-2′-deoxyuridine incorporation with click chemistry, an alternative to using 5-bromo-2′-deoxyuridine antibodies. *BioTechniques*.

[3] Cappella P., Gasparri F., Pulici M., Moll J., Robinson J. P. (2008). UNIT 7.34 cell proliferation method: click chemistry based on BrdU coupling for multiplex antibody staining. *Current Protocols in Cytometry*.

[4] Du Y., Kong G., You X. (2012). Elevation of highly up-regulated in liver cancer (HULC) by hepatitis B virus X protein promotes hepatoma cell proliferation via down-regulating p18. *Journal of Biological Chemistry*.

[5] Ge Y.-F., Sun J., Jin C.-J., Cao B.-Q., Jiang Z.-F., Shao J.-F. (2013). AntagomiR-27a targets FOXO3a in glioblastoma and suppresses U87 cell growth in vitro and in vivo. *Asian Pacific Journal of Cancer Prevention*.

[6] Qiu J., He Y., Wang Y. (2013). Plumbagin induces the apoptosis of human tongue carcinoma cells through the mitochondria-mediated pathway. *Medical Science Monitor Basic Research*.

[7] Yue L., Li L., Liu F. (2013). The oncoprotein HBXIP activates transcriptional coregulatory protein LMO4 via Sp1 to promote proliferation of breast cancer cells. *Carcinogenesis*.

[8] Ohyama W., Okada E., Fujiishi Y., Narumi K., Yasutake N. (2013). In vivo rat glandular stomach and colon micronucleus tests: kinetics of micronucleated cells, apoptosis, and cell proliferation in the target tissues after a single oral administration of stomach- or colon-carcinogens. *Mutation Research*.

[9] Jeong K. C., Kim K. T., Seo H. H. (2014). Intravesical instillation of c-MYC inhibitor KSI-3716 suppresses orthotopic bladder tumor growth. *Journal of Urology*.

[10] Jiang B., Li Z., Zhang W. (2014). miR-874 Inhibits cell proliferation, migration and invasion through targeting aquaporin-3 in gastric cancer. *Journal of Gastroenterology*.

[11] Dong S., Qu X., Li W. (2015). The long non-coding RNA, GAS5, enhances gefitinib-induced cell death in innate EGFR tyrosine kinase inhibitor-resistant lung adenocarcinoma cells with wide-type EGFR via downregulation of the IGF-1R expression. *Journal of Hematology & Oncology*.

[12] Guo J., Li D., Bai S., Xu T., Zhou Z., Zhang Y. (2012). Detecting DNA synthesis of neointimal formation after catheter balloon injury in GK and in Wistar rats: using 5-ethynyl-2′-deoxyuridine. *Cardiovascular Diabetology*.

[13] Amano S. U., Cohen J. L., Vangala P. (2014). Local proliferation of macrophages contributes to obesity-associated adipose tissue inflammation. *Cell Metabolism*.

[14] Cohen J. E., Purcell B. P., MacArthur J. W. (2014). A bioengineered hydrogel system enables targeted and sustained intramyocardial delivery of neuregulin, activating the cardiomyocyte cell cycle and enhancing ventricular function in a murine model of ischemic cardiomyopathy. *Circulation: Heart Failure*.

[15] Zeng B., Tong S., Ren X., Xia H. (2014). Cardiac cell proliferation assessed by EdU, a novel analysis of cardiac regeneration. *Cytotechnology*.

[16] Tsiperson V., Huang Y., Bagayogo I. (2015). Brain-derived neurotrophic factor deficiency restricts proliferation of oligodendrocyte progenitors following cuprizone-induced demyelination. *ASN Neuro*.

[17] Budhiraja R., Tuder R. M., Hassoun P. M. (2004). Endothelial dysfunction in pulmonary hypertension. *Circulation*.

[18] Montani D., Price L. C., Dorfmuller P. (2009). Pulmonary veno-occlusive disease. *European Respiratory Journal*.

[19] Mandel J., Mark E. J., Hales C. A. (2000). Pulmonary veno-occlusive disease. *American Journal of Respiratory and Critical Care Medicine*.

[20] Laveneziana P., Montani D., Dorfmuller P. (2014). Mechanisms of exertional dyspnoea in pulmonary veno-occlusive disease with EIF2AK4 mutations. *European Respiratory Journal*.

[21] Perros F., Cohen-Kaminsky S., Gambaryan N. (2013). Cytotoxic cells and granulysin in pulmonary arterial hypertension and pulmonary veno-occlusive disease. *American Journal of Respiratory and Critical Care Medicine*.

[22] Eyries M., Montani D., Girerd B. (2013). EIF2AK4 mutations cause pulmonary veno-occlusive disease, a recessive form of pulmonary hypertension. *Nature Genetics*.

[23] Ranchoux B., Günther S., Quarck R. (2015). Chemotherapy-induced pulmonary hypertension: role of alkylating agents. *The American Journal of Pathology*.

[24] Montani D., Kemp K., Dorfmuller P., Sitbon O., Simonneau G., Humbert M. (2009). Idiopathic pulmonary arterial hypertension and pulmonary veno-occlusive disease: similarities and differences. *Seminars in Respiratory and Critical Care Medicine*.

[25] Perros F., Günther S., Ranchoux B. (2015). Mitomycin-induced pulmonary veno-occlusive disease: evidence from human disease and animal models. *Circulation*.

[26] Ranchoux B., Antigny F., Rucker-Martin C. (2015). Endothelial-to-mesenchymal transition in pulmonary hypertension. *Circulation*.

[27] Izikki M., Mercier O., Lecerf F. (2013). The beneficial effect of suramin on monocrotaline-induced pulmonary hypertension in rats. *PLoS ONE*.

[28] Sun Y., Lin G., Zhang R. (2012). Multicolor flow cytometry analysis of the proliferations of T-lymphocyte subsets in vitro by EdU incorporation. *Cytometry Part A: Journal of the International Society of Analytical Cytology*.

[29] Zeng C., Pan F., Jones L. A. (2010). Evaluation of 5-ethynyl-2′-deoxyuridine staining as a sensitive and reliable method for studying cell proliferation in the adult nervous system. *Brain Research*.

[30] Neumann P., Koch S., Hilgarth R. S. (2014). Gut commensal bacteria and regional Wnt gene expression in the proximal versus distal colon. *The American Journal of Pathology*.

[31] Andersen D. C., Skovrind I., Christensen M. L., Jensen C. H., Sheikh S. P. (2013). Stem cell survival is severely compromised by the thymidineanalog EdU (5-ethynyl-2′-deoxyuridine), an alternative to BrdU for proliferation assays and stem cell tracing. *Analytical and Bioanalytical Chemistry*.

[32] Asano M., Yamamoto T., Tsuruta T., Nishimura N., Sonoyama K. (2015). Dual labeling with 5-bromo-2'-deoxyuridine and 5-ethynyl-2′-deoxyuridine for estimation of cell migration rate in the small intestinal epithelium. *Development, Growth & Differentiation*.

