# Corrigendum to “A Simple Method to Assess *In Vivo* Proliferation in Lung Vasculature with EdU: The Case of MMC-Induced PVOD in Rat”

**DOI:** 10.1155/2017/4697379

**Published:** 2017-02-06

**Authors:** Fabrice Antigny, Benoît Ranchoux, Valérie Nadeau, Edmund Lau, Sébastien Bonnet, Frédéric Perros

**Affiliations:** ^1^Faculté de Médecine, Université Paris-Sud, Le Kremlin-Bicêtre, France; ^2^AP-HP, Centre de Référence de l'Hypertension Pulmonaire Sévère, Département Hospitalo-Universitaire (DHU) Thorax Innovation, Service de Pneumologie et Réanimation Respiratoire, Hôpital de Bicêtre, Le Kremlin-Bicêtre, France; ^3^UMRS 999, INSERM and Université Paris-Sud, Laboratoire d'Excellence (LabEx) en Recherche sur le Médicament et l'Innovation Thérapeutique (LERMIT), Centre Chirurgical Marie Lannelongue, Le Plessis-Robinson, France; ^4^Groupe de Recherche en Hypertension Pulmonaire, Centre de Recherche de l'Institut Universitaire de Cardiologie et de Pneumologie de Québec, QC, Canada

In the article titled “A Simple Method to Assess In Vivo Proliferation in Lung Vasculature with EdU: The Case of MMC-Induced PVOD in Rat” [1], the authors' first and last names were reversed. The corrected authors' list is shown above.
